# Capture and Ex-Situ Analysis of Environmental Biofilms in Livestock Buildings

**DOI:** 10.3390/microorganisms10010002

**Published:** 2021-12-21

**Authors:** Virgile Guéneau, Ana Rodiles, Jean-Christophe Piard, Bastien Frayssinet, Mathieu Castex, Julia Plateau-Gonthier, Romain Briandet

**Affiliations:** 1 Micalis Institute, INRAE, AgroParisTech, Université Paris-Saclay, 78350 Jouy-en-Josas, France; virgile.gueneau@inrae.fr (V.G.); jean-christophe.piard@inrae.fr (J.-C.P.); 2 Lallemand SAS, 31702 Blagnac, France; arodiles@lallemand.com (A.R.); bfrayssinet@lallemand.com (B.F.); mcastex@lallemand.com (M.C.); jplateau@lallemand.com (J.P.-G.)

**Keywords:** biofilm, sampling, livestock building, surfaces, diversity

## Abstract

Little information about biofilm microbial communities on the surface of livestock buildings is available yet. While these spatially organized communities proliferate in close contact with animals and can harbor undesirable microorganisms, no standardized methods have been described to sample them non-destructively. We propose a reproducible coupon-based capture method associated with a set of complementary ex-situ analysis tools to describe the major features of those communities. To demonstrate the biofilm dynamics in a pig farm building, we analyzed the coupons on polymeric and metallic materials, as representative of these environments, over 4 weeks. Confocal laser scanning microscopy (CLSM) revealed a rapid coverage of the coupons with a thick layer of biological material and the existence of dispersed clusters of active metabolic microorganisms. After detaching the cells from the coupons, counts to quantify the CFU/cm^2^ were done with high reproducibility. High-throughput sequencing of the 16S rRNA V3-V4 region shows bacterial diversity profiles in accordance with reported bacteria diversity in pig intestinal ecosystems and reveals differences between materials. The coupon-based methodology allows us to deepen our knowledge on biofilm structure and composition on the surface of a pig farm and opens the door for application in different types of livestock buildings.

## 1. Introduction

In intensive breeding farms, animals live in a confined environment directly on inorganic floors (slatted floors, cages, aviary) or organic litter (straw, sawdust). In these environments, animal density can be very high, and the running parameters of farms (animal nutrition, temperature, humidity, light) are modulated to obtain their maximal growth rate. Concrete, metals, and polymers composed the majority of housing equipment materials in direct contact with animals in the livestock buildings, such as water and feed distribution systems, fences, pens, cages, walls, or gratings [1]. To reduce organic matter and microbial development on these surfaces, cleaning and disinfection (C&D) procedures are performed between two batches of animals, as well as regular removal of manure during the batch. However, these procedures are far from eradicating sessile microflora that can harbor pathogenic subpopulations [2,3,4]. Indeed, it is estimated that between 40 and 80% of living microorganisms are associated with a surface in so-called biofilms, which are present in all biotopes on earth [5]. Biofilms are three-dimensional biological structures composed of microbial communities embedded in cohesive self-produced extracellular polymeric substances (EPS) [6]. EPS can drastically vary between biofilms but is generally composed of water and a complex mixture of polysaccharides, extracellular DNA (eDNA), proteins, and amyloid fibers. The presence of EPS, along with spatial organization and specific signaling systems, triggers a diversification of cell types and emerging community functions, including a fantastic adaptation to environmental fluctuations and the action of antimicrobials, in comparison to their free planktonic homologs [7,8,9,10,11,12].

Because of spatial proximity and high cell density, biofilms are considered as hot spots for the spreading of antibiotic resistance genes and virulence factors by horizontal gene transfer [13,14,15]. 

Current knowledge about surface-associated microbial communities in livestock buildings is still very limited, while of prime importance to decipher microbial pathogens dynamics in these environments and their interactions with animals. These questions enter in the One Health context by pinpointing the flow of pathogens between the environment, animals, and humans. Furthermore, national and international regulations are evolving to limit the use of antimicrobials such as antibiotics (to prevent antibiotic resistance) and disinfectants (to prevent environmental pollution) [16]. The development of innovative and alternative strategies are hence explored and are needed to create scientific knowledge and methodologies to analyze complex multispecies biofilms on livestock building surfaces and their dynamics under perturbations [17,18]. 

In farms, most pathogenic microorganisms responsible for zoonosis can be associated and protected within environmental biofilms. Holobiont of animals is in constant interplay with the biofilms from the farm environment. These biofilms may constitute an environmental route for animal and human contamination [19]. Quantification of undesirable species such as bacterial pathogens is hence carried out in livestock buildings regularly to monitor its hygienic status and to comply with eventual national or international regulations. The sampling methodologies used are principally swabs, sponges, and contact plates with specific media [20,21]. Nowadays, there is no chemical or physical methodology able to extract all surface-associated communities from a surface to study it [22,23], and the extracted fraction may not be representative of the initial sessile community [24]. Another strong limitation of these sampling methods is the definitive loss of the biofilm spatial organization that is, as mentioned previously, a major factor of microbial persistence on surfaces. 

Indeed, protocols with coupons, defined here as a small surface of a specific material where the biofilm can develop, have been designed and used to capture in-situ and non-invasively the microbial community of interest in other systems [25]. These types of sampling methods were in particular successively applied to describe biofilms in aquatic conditions (biocorrosion in the sea, drinking water distribution system, and wastewater treatment) [26,27,28,29]. Coupons have also been recently used to detect the pathogenic bacteria *Listeria monocytogenes* in the food processing industry [30]. The coupon-based method allows structural analysis of the microbial community using microscopy techniques that can be combined with microbial diversity and bacterial counts. Moreover, it has been shown that biofilms growing on coupons are representative of the surface of the surroundings and that this method is more robust than classical environmental swabbing with less variability in bacterial counting [31]. 

In this study, we implemented a coupon-based methodology to capture native biofilms on livestock building surfaces (here a pig farm) along with a set of ex-situ analyses to describe the structural dynamics of these communities over one month. CLSM analysis was put in use to decipher non-invasively the three-dimensional structure of the biofilm, with a special interest in contrasting metabolically active cells. Bacterial plate counting was performed on non-selective media to allow quantification of viable and cultivable species, while an analysis of the bacterial diversity was performed by high-throughput sequencing of the 16S rRNA V3-V4 regions.

## 2. Materials and Methods

### 2.1. Livestock Building, Coupons Disposition, and Sampling

The coupons were placed in a French commercial pig farm during the post-weaning stage of piglets over 31 days (Blan, France). The livestock building was a slatted floor pen system. The temperature was 27 °C at animals’ entry with a decrease of 1 °C every week to reach 24 °C until the end of breeding. Representative coupons of livestock building surface materials were composed of polyvinyl chloride (PVC) or galvanized steel (steel) with dimensions: 2.5 cm × 6 cm × 3 mm (Leroy-Merlin, Colomiers, France). Coupons were sterilized in an autoclave (HMC EUROPE, Tuslin, Germany) and dried in a dry oven (FD 115 model, Binder, Tuttlingen, Germany) for 15 min at 120 °C.

**Figure 1:**
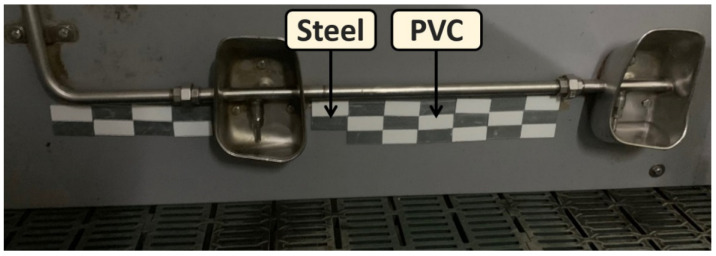
Arrangement of coupons on the wall. Steel and PVC coupons were placed on the walls under the water lines using double-sided tape in a staggered arrangement.

In this work, 60 coupons of each material were analyzed (120 coupons in total) side by side on pen dividers using double-sided tape under the water lines close to animals in five pens of 3.73 m per 2.5 m of 25 piglets each (Figure 1). 

Per sampling day and in the same way for the 5 pens, 2 coupons of each material were extracted, one for microscopy study and the second one to perform the bacterial counts and DNA extraction for high-throughput sequencing analysis. The beginning of the experiment (day 0) corresponds to coupons implantation on pen dividers, after cleaning using the DECABAZ (HYDRACHIM, Étrelles, France) detergent and the VIROCID (CID lines, Ypres, Belgium) for the disinfection step, following manufacturer recommendations. A sampling of the coupons was made from the outside to the inside of the coupon line on days 2, 6, 7, 14, 21, and 31. The first sampling point on day 2 is just before the entry of animals. During sampling, coupons were aseptically removed with gloves from the pen dividers and placed in Petri dishes containing a sterile compress soaked in sterile water to avoid sample dehydration until analysis (<24 h after sampling).

### 2.2. Confocal Laser Scanning Microscopy

Biofilm structures on coupons were observed using a high-content screening confocal laser scanning microscope (LEICA, HCS-SP8, Wetzlar, Germany) at the INRAE MIMA2 microscopic platform (www6.jouy.inrae.fr/mima2_eng, accessed on 10 December 2021). Then, 50 µL of a 54 µM calcein acetoxymethyl (CAM), a metabolic fluorescent dye reporting esterase activity in green, was poured on the coupons and incubated in the dark for 30 min at 37 °C (Invitrogen, Carlsbad, CA, USA). The non-ionic molecules can enter passively into cells and be cleaved by intracellular esterase, releasing a fluorescent non-permeant ionic residue. Biofilms on the coupons were also counter labeled in red with 50 µL of a 3 µL/mL of SYTO 61 (Invitrogen, Carlsbad, CA, USA), a cell permanent red dye that labels nucleic acid. 

A 600 Hz frequency was used to acquire images with the CLSM. SYTO 61 was excited with the HeNe laser at 633 nm, and the emitted fluorescence was collected with a hybrid detector in the range from 650 to 700 nm. CAM was excited with an argon laser set at 488 nm, and the emitted fluorescence was collected with a hybrid detector in the range from 500 to 550 nm. The surface topography was also captured using the reflection mode of the CLSM with the 488 nm laser line. A series of 512 × 512 pixels images was acquired using a 63× water lens (numerical aperture = 1.2) for the first samples (day 2) and a 40× air lens (numerical aperture = 0.8) for the other thick samples by taking one image per µm in the *z*-axis to capture the full height of the biofilm. 2D projections and image analysis were performed using IMARIS 9.3.1 software (Bitplane, AG—Zurich, Switzerland).

Quantification of fluorescence signals was performed with the two labels to determine the evolution of each compartment (biovolume in µm^3^/µm^2^) over time for the two materials.

### 2.3. Enumeration of Bacteria Detached from Coupons

Coupons were placed in individual tubes containing 30 mL of a saline solution (NaCl 9 g/L) to cover halfway. With a sterile pipette tip, the biofilm was mechanically disrupted by successive round trips corresponding to 20 vertical and horizontal passages on the immersed side of the coupon, and then, the latter was turned over to do the same on the other side. Then, removing the coupon and suspending the bacteria by vortexing for 5 s the liquid, successive dilutions in saline solution were carried out in duplicate using 1mL of the resuspended biofilm solution. Counts into agar were made from 1 mL of the desired dilution. Trypticase soy broth with agar (TSA (*w/v*); 1% tryptone, 0.5% yeast extract, 0.5% NaCl, 1.5% agar; BioMérieux, Marcy-l’Étoile, France) was used as non-selective media under aerobic conditions for 24 h at 30 °C. To select the spores, 1mL of the biofilm solution was placed in a glass tube, in a water bath for 10 min at 80 °C, in duplicate for each coupon before enumeration by TSA. The Petri dishes in a CFU range between 30 and 300 were counted. The remaining 26 mL of the detached biofilm solution was centrifuged for 10 min at 6000× *g*, the supernatant was gently removed, and the pellets were placed at −20 °C to extract after the DNA.

### 2.4. High-Throughput Sequencing of the 16S rRNA and Diversity Analysis

#### 2.4.1. DNA Extraction, PCR, and Sequencing

DNA from 60 bacterial pellets were extracted and purified using DNeasy PowerLyzer PowerSoil Kit manufacturer instructions (Qiagen, Hilden, Germany). PCR of V3–V4 regions of 16S rRNA marker genes was carried out on a thermocycler (Geneamp PCR system 9700, Applied Biosystems, USA) with universal primers F343 (5-CTTTCCCTACACGACGCTCTTCCGATCTTACGGRAGGCAGCAG-3) and R784 (5-GGAGTTCAGACGTGTGCTCTTCCGATCTTACCAGGGTATCTAATCCT-3) with an annealing temperature of 66 °C using Phusion High-Fidelity PCR kit (New England Biolabs, Ipswich, MA, USA) [32]. DNA was quantified on a NanoDrop Spectrophotometer ND-1000 (Thermo Fisher, Waltham, MA, USA). PCR products, including negative controls, were checked on 1% agarose gel electrophoresis to ensure PCR products. Illumina Miseq technology was used to sequence the amplicons (GeT-PlaGe INRAE platform, Toulouse, France).

#### 2.4.2. Diversity Analysis Using Bioinformatics

Paired-end Fastq files were denoised with DADA2 [33] by default parameters, including consensus chimeras removal, as well removing primers and truncation by Demux with a final length of 411 ± 26 bp. Multiple alignment using fast Fourier transform (MAFFT) was used to perform de novo multiple sequence alignments [34], and mask as gap filtering Phylogeny was constructed with FastTree [35,36]. Data were rarefied to 10,120 sequences per sample; then low abundance amplicon sequence variants (ASVs) per pen were filtered out (<100 seqs in 2 samples, to improve accuracy on diversity estimates [37]). Rarefaction curves as observed AVSs and good coverage were studied to ensure a full sampling of the community was taken. Alpha diversity parameters (Shannon) and richness (Observed ASVs) were compared across materials per sampling point, as well as weighted UniFrac distances for Beta diversity. All bioinformatics results and graphs were obtained in QIIME2, using the Python-based microbiome data-science platform [38].

### 2.5. Statistical Analysis

Results are represented by the average and standard deviation (SD) or confidence interval (CI) of 5 coupons per day, and day was considered the experimental unit. Two-way ANOVA using the uncorrected Fisher’s least was used for the count and biovolume analysis with PRISM software (GraphPad, San Diego, California, USA). Taxonomy data were analyzed with Lefse [39] with default parameters, alpha diversity with Kruskal–Wallis, and beta diversity PERMANOVA in QIIME2 [38]. Data were considered significant when a *p*-value were smaller than 0.05.

## 3. Results

### 3.1. Coupons Are Colonized by a Densely Clustered Biofilm with Only a Minor Fraction of Cells Metabolically Active

**Figure 2:**
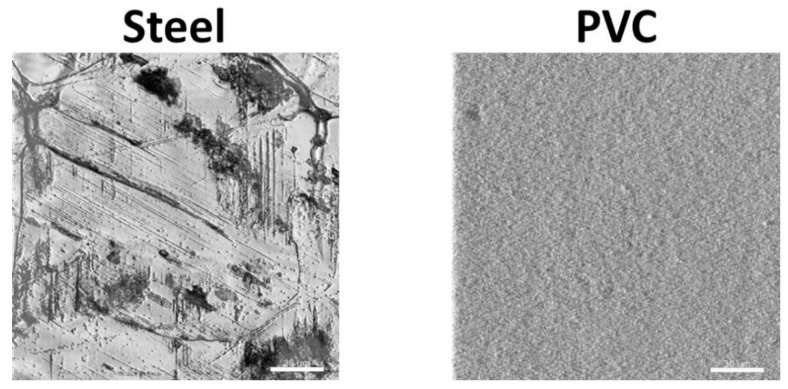
Characterization of the surface of coupons using confocal laser scanning microscopy. A detail of the surface topography of the steel and the PVC coupons by CLSM are shown (scale bar = 30 µm).

Before being placed in the farm, coupons surface topography was analyzed with the reflection mode of a CLSM (Figure 2). The surface of steel was rougher than PVC with the presence of streaks and holes in abundance, while the PVC was very smooth. To estimate the hydrophilicity of the coupons, contact angles with water were measured [40]. The two side contact angles of 15 drops of 100 µL were measured with the image analysis software ImageJ (1.53 version). The water contact angles show that steel coupons (angle of 51.1° ± 7.4) were more hydrophilic (*p* < 0.05) than PVC coupons (angle of 84.8° ± 3.2).

**Figure 3:**
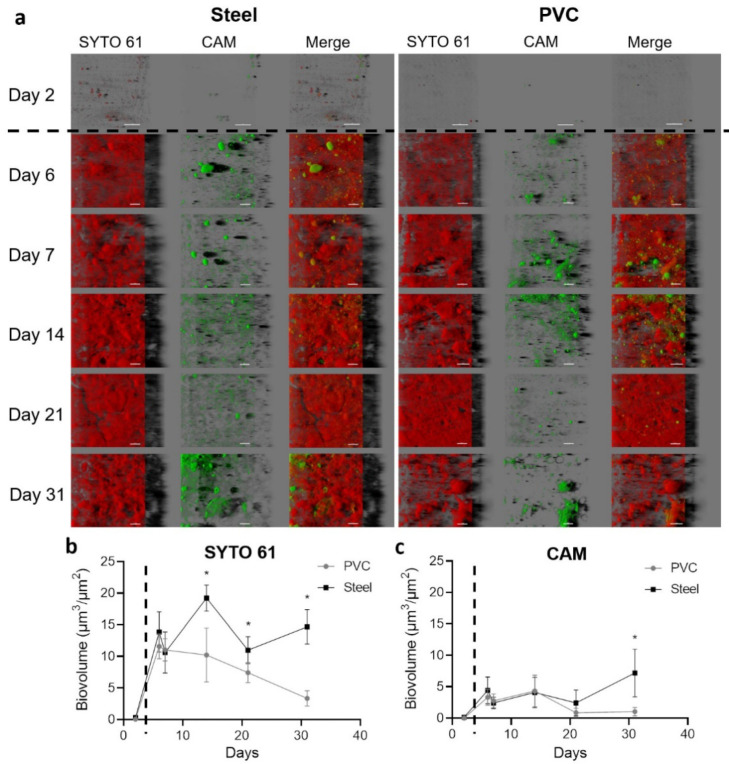
Confocal laser scanning microscopy visualization of biofilms settled on the steel and PVC coupons. Microorganisms and eDNA were labeled in red with SYTO61 and metabolically active cells in green with CAM. The easy 3D representative projections for each time point for steel and PVC coupons are shown (**a**). Day 2 corresponds to the first sampling, two days after the deposit of the coupons without animals in the farm; the scale bar represents 30 µm for day 2 and 40 µm for the other time points. The biovolume of SYTO 61 (**b**) and CAM (**c**) signals were extracted over time on PVC (grey lines) and steel (black lines). The dotted line indicates the animal’s entry into the building. Results represent average ±CI 95% (* p < 0.05).

Coupons harvested in the building were labeled with SYTO 61 (red) to mark intra- and extracellular nucleic acids in the biofilms and CAM (green) to highlight metabolically active microorganisms inside the community (Figure 3a). 

Images of the first sampling day after the cleaning and disinfection process and before the entry of animals in the farm (day 2) showed only a few sessile scattered microorganisms with the size around the micrometer compatible with bacteria. After animals entered, biovolumes in both channels sharply and significantly increased (*p* < 0.05) (Figure 3b). From sampling day 6 until the end of the experiment, material contrasted with SYTO 61 was covering all the coupons surfaces. An organization with clusters or compact structures with holes in both materials was visualized. The biovolume of SYTO 61-labeled material was significantly higher on steel than PVC from day 14, and a decrease of the signal was observed from day 7 in PVC (*p* < 0.05).

Green CAM-labeled clusters corresponding to metabolically active microorganisms were observed in all samples after animal entry. Biovolume of CAM was higher on steel in comparison to PVC on day 31 (*p* < 0.05), and a decrease of biovolume appeared for days 21 and 31 on PVC (*p* < 0.05) (Figure 3c).

### 3.2. Enumeration of Aerobic Cultivable Bacteria from Coupons

**Figure 4:**
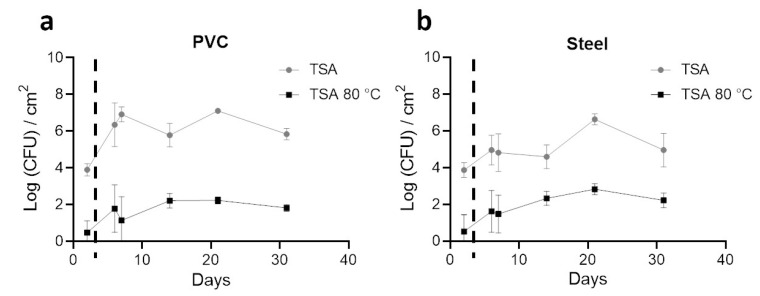
Enumeration profiles (Log CFU/cm^2^) of the total aerobic bacteria (TSA) and spores (TSA, 80ºC, 10min) on steel and PVC coupons during the trial. Biofilms on coupons were removed mechanically by pipetting and by a round trip with saline solution. After successive dilutions, bacteria inside samples were enumerated on TSA (grey lines) and on TSA after a 10 min at 80 °C treatment to select spores (black lines). Results are average ±CI 95% of 2 enumeration profiles for PVC (**a**) and Steel (**b**) coupons. The dotted line indicates the animal’s entry into the building.

Enumeration with TSA plates as a non-selective media to quantify total aerobic bacteria on coupons was performed. In addition, heat treatment of 10 min at 80 °C was carried out before TSA plating to select spores from the same samples (Figure 4). 

Before the entry of animals and 2 days after coupon placement (day 2), 4 logs (CFU/cm^2^) of total bacteria were enumerated in both materials. After animals entered, a significant increase of total aerobic cultivable bacteria was obtained on PVC in comparison to steel (*p* < 0.05) for all days, except at day 21. A stabilization of non-selective counting was obtained after animals entered with a value around 6 logs (CFU/cm^2^) for PVC and 5 logs (CFU/cm^2^) for steel. A peak was observed in both materials on day 21. With heat treatment, less than 1 log (CFU/cm^2^) of spores were enumerated on both materials on the first sampling. Values increased on PVC and steel after day 2 to reach more than 2 logs (CFU/cm^2^) of spores on day 21; no significant differences were observed on both materials for each time point. A higher number of total aerobic cultivable bacteria were counted significantly on PVC compared to steel (*p* < 0.05), however, with a tendency to have fewer spores counted.

### 3.3. 16S rRNA High-Throughput Sequencing Analysis to Decipher the Dynamic of Biofilm Bacterial Diversity

**Figure 5:**
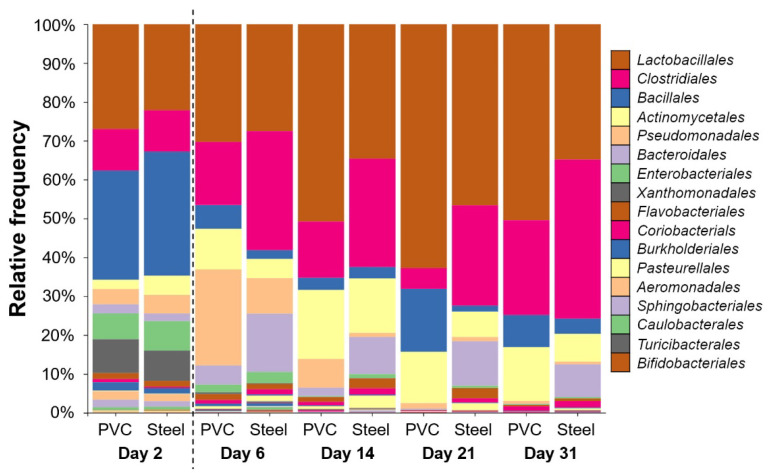
Taxonomic order profile of bacterial communities on the pig farm surface determined using PVC and steel coupons. Each color bar represents the relative frequency of one bacterial order inside the total bacterial community. For each day, PVC and steel taxonomic profiles are shown. The dotted line indicates the animal’s entry into the building.

A total of 60 samples from PCR targeting the 16S rRNA coding gene were successfully sequenced. After error filtering, alignment, and chimera removal, 1,146,915 reads were generated, corresponding to 19,115 ± 6505 sequences per sample. Taxonomic analysis shows that *Firmicutes* phylum was the most represented in all samples, followed by *Proteobacteria*, *Actinobacteria*, and *Bacteroidetes*. *Lactobacillales*, *Clostridiales*, *Bacillales*, *Actinomycetales*, *Pseudomonadales*, *Bacteroidales*, and *Enterobacteriales* were the most dominant orders (Figure 5). 

*Lactobacillales* was the most represented order in both materials when samples were compiled for days with significantly more relative frequency on PVC (40%) compared to steel (31%). The trends are reversed with *Clostridiales* that is the second most represented significantly on steel (25%) compared to PVC (14%) (*p* < 0.05). Other orders were also significantly different per material: *Bacillales*, *Actinomycetales*, and *Pseudomonadales* on PVC and *Bacteroidales*, *Rhodocyclales*, *Enterobacteriales*, *Coriobacteriales*, and *Flavobacteriales* on steel (*p* < 0.05). The trends stated above are true for each time point except for day 2 (Figure 4). On day 2, after C&D procedures and before the animals enter the building, taxonomic profiles are different, with the larger abundance corresponding to *Bacillales* order on both materials (28–32%). Just after animals enter at day 6, *Pseudomonadales* order proportion increases to reach 25% of relative frequency on the PVC in comparison to a less increase on the steel of 9%. *Bacteroidales* are more detectable on steel than PVC for every time point except for day 2, which is at the same level. The level of *Enterobacteriales* decreased from day 2 to the end of the experiment (Figure 5). 

**Figure 6:**
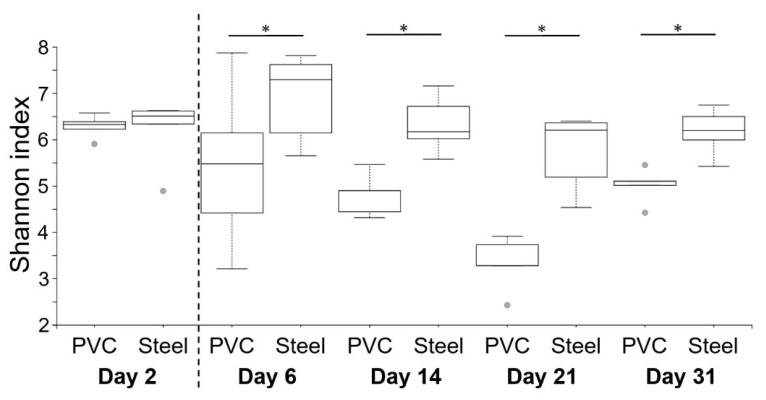
Comparison of α-diversity on PVC and steel coupons during the experiment. α-diversity was determined using the Shannon index value. The dotted line indicates the animal’s entry into the building. Pairwise comparisons between materials per day are shown with an asterisk (* *p* < 0.05).

Shannon indexes were used to compare α-diversity between steel and PVC materials during the experiment (Figure 6). On day 2, before animal entry, the diversity was the same on both materials (6.3 and 6.2). The Shannon index on PVC decreased on day 6 until day 21, in comparison to a significantly higher and stable diversity level on steel for all the breeding duration. Except for the first sampling on day 2, a significantly lower level of diversity is observed on PVC compared to steel (*p* < 0.05), corresponding to a decrease of richness and evenness in the bacterial population. Similar results were found for observed ASVs from 285 at day 2 to a peak on day 6 with 341–431, reaching 170–290 ASVs at the end of the trial, for PVC and steel, respectively. Weighted UniFrac distances of beta diversity showed significant differences between the compositional 16S of both materials per day (*p* < 0.05).

## 4. Discussion

Knowledge of the microbial communities living on surfaces of livestock buildings is still very limited in the literature. These communities, in close contact with animals, should be better characterized for their control. As mentioned previously, no standard or robust methodology is described to sample or analyze these spatially organized communities. Here, we propose a sampling and analyzing method of the biofilms by collecting them from coupons installed on site.

To apply and validate this methodology in the context of pig farms, coupons were placed after C&D protocol under the water lines, an area with frequent contact with animals. Coupons were sampled before animal entry that corresponds to 2 days of incubation (day 2), and around 4 logs (CFU/cm^2^) of bacteria were counted on TSA with both materials. After several days, the number of counts did not reach more than 7 logs (CFU/cm^2^), showing low effectiveness of C&D protocol or rapid growth of bacteria after C&D. Analysis of coupons incubated throughout the breeding period exposed or not to a C&D process would allow answering this question.

The spatial organization of metabolically active bacteria inside the total biofilm was observed. By compiling the biovolume of both channels over time and by the ratio of CAM versus SYTO61, it was possible to estimate that for the two materials, around 30% of the biofilm was composed of metabolically active cells. The bacteria counted on TSA medium may be those detected by CAM without the viable but non-cultivable part. Metabolically active bacteria in the biofilm can have a commensal origin or can be those detected on surfaces before entry of animals which have developed, representing in our study around 4 logs (CFU/cm^2^) for day 2, and which are by diversity analysis principally *Bacillales* and *Lactobacillales*. While CAM allowed the visualization of localized pockets of metabolically active cells, a limitation of such esterase activity markers is that some bacterial species can expulse the dye with efflux pumps, resulting in loss of intracellular fluorescence [41].

Here, coupons were contaminated with feces splashes from the arrival of the animals in the building (day 6). Fecal bacteria can hence be recruited in the preexisting biofilm and be integrated into the CFU counts. It has been shown that the bacterial quantity that lives in the gut achieves among the highest cell densities recorded for any ecosystem, with more than 10^11^ cells per gram of wet material in the colon [42]. The intestinal contents of pig harbor a fraction of strict microbial anaerobes that do not tolerate the presence of oxygen. Their release into open air can cause cell death and lysis and the release of eDNA [43]. In this study, the SYTO 61 can label live and dead microorganisms but also the abundant eDNA fraction in the matrix. Previous studies have shown that polysaccharides, proteins, and eDNA can play a role in the spatial structure of the biofilm [6,44]. The very compacted structure of biofilms may here be linked to the large amount of eDNA resulting from bacterial lysis.

A decrease in SYTO 61 signal on PVC is observed from day 6 to the end of the experiment. Biofilms on PVC could be less cohesive than on steel. The steel coupons showed high hydrophilic properties and harbored holes and cracks on their surface that could permit a better fixation of organic materials in comparison to PVC. The structure of the biofilm changed with a decrease of the biomass level during the experiment, but this was not correlated with a decrease in bacterial number. Biovolumes on steel were higher than on PVC, and this could also be explained by the properties of steel surface (hydrophobicity and rugosity). However, the number of bacteria counted on PVC was higher than on steel, which suggests that steel carries a greater matrix with fewer bacteria. Microscopic visualization of extracellular matrix compounds may be considered in future experiments. Alternative labeling methods can be used in future experiments to distinguish other components of the biofilm, such as fluorescent lectins that bind specifically to exopolysaccharides, thioflavin T for amyloid fiber labeling, or fluorescence in situ hybridization (FISH) technique by detecting specific species having access to their spatial organization [45,46]. 

The bacterial diversity was studied by 16S high-throughput sequencing analysis. Overall, in the absence of additional biological replicates, the insights brought by 16S sequencing were limited to the description of the microbial community. The results showed more richness and evenness in steel than PVC, and this was in line with the taxonomy results, with the presence of a large proportion of bacteria on coupons, such as *Lactobacillales*, *Clostridiales*, *Bacillales*, *Actinomycetales*, *Pseudomonadales*, *Bacteroidales*, and *Enterobacteriales*, already found in other pig studies [47,48,49]. A bias in this community profiling arises from the high quantities of bacterial eDNA from feces on the surface that can be sequenced in addition to the living bacteria that multiply in such biofilms. DNA of bacteria that can grow on the surface was amplified, but because of the eDNA release, dead bacteria are also detected with this approach. V3–V4 regions of the gene encoding the 16S RNA were chosen for DNA amplification to discriminate better the species as bringing higher variability of the amplified sequences, in comparison to other regions like V4–V5 region, which can show as well the presence of archaea [50]. Internal transcribed spacer (ITS) regions from fungi that code for ribosomal RNA can also be used to study this diversity of surface communities [51]. A probable correlation exists between the composition of the animal microbiota and the microbial communities identified on the coupons. For future experiments, animal microbiota has to be sampled, analyzed, and compared to the biofilms.

This method has been validated in a pig farming context, allowing a better understanding of the dynamics of microbial communities over time in this production system. In the future, we could envision that a farmer could use this coupon method without the intervention of specialized technician and send them to the laboratory with a dedicated procedure for monitoring the building surface microbiological profile. Interesting trends such as the effect of the material stand out significantly and should be extended by other complementary studies in other farms to standardize the results. The method also makes it possible to isolate strains of interest from these surfaces. New high-throughput culturomic techniques compatible with our sampling methodology allow us to isolate and cultivate viable bacteria inside the community and could help to avoid the bias surface contamination by dead bacteria from feces that are detected by DNA sequencing [52,53,54]. In perspective, a study where coupons are placed at different heights to also study the formation of biofilm on these surfaces without splashes of feces could be considered. Other coupons composed of representative materials of the livestock building as concrete or stainless steel could also be analyzed. 

Antimicrobial tolerance and resistance of such compact 3D communities will be also interesting to investigate. These microbial communities are also frequently exposed to antibiotic residues and can become reservoirs of antibiotic resistance genes.

## 5. Conclusions

A method to non-invasively capture biofilms of the surface of livestock buildings and their ex-situ analysis was developed in this work to be implemented and validated in a building of a pig farm. Bacterial density, diversity, and the three-dimensional structure of the surface biofilms were analyzed, thus increasing our knowledge of these microbial communities, poorly documented until now. In perspective, these results could contribute to better understanding the contamination risks of animals by pathogens present on the contact surfaces and directly associated with biofilms. In the future, this approach could be used to study surface biofilms in a variety of agricultural environments, including other types of livestock buildings.
